# Parasite-driven pathogenesis in *Trypanosoma brucei* infections

**DOI:** 10.1111/j.1365-3024.2011.01286.x

**Published:** 2011-08

**Authors:** L J Morrison

**Affiliations:** Wellcome Trust Centre for Molecular Parasitology, Institute of Infection, Immunity and Inflammation, College of Medical, Veterinary and Life Sciences, University of GlasgowGlasgow, UK

**Keywords:** innate immunity, nagana, pathogenesis, sleeping sickness, trypanosome

## Abstract

Trypanosomes are protozoan parasites of medical and veterinary importance. It is well established that different species, subspecies and strains of trypanosome can cause very different disease in the mammalian host, exemplified by the two human-infective subspecies of *Trypanosoma brucei* that cause either acute or chronic disease. We are beginning to understand how the host response shapes the course of the disease and how genetic variation in the host can be a factor in disease severity, particularly in the mouse model, but until recently the role of parasite genetic variation that determines differential disease outcome has been a neglected area. This review will discuss the recent advances in this field, covering both our current knowledge of the *T. brucei* genes involved and the approaches that are leading towards the identification of *T. brucei* virulence genes. Finally, the potential for using parasite genotype variation to examine the evolutionary context of virulence will be discussed.

## Introduction

Trypanosomes are single-celled protozoa, transmitted by tsetse flies (*Glossina* spp.) to a wide range of mammalian hosts. They are of well-documented medical significance to both humans and livestock (1,2). It has been a long established focus of trypanosome research that the genetic background of the host plays an important part in determining disease outcome, and this has formed the basis of a formidable body of work aiming to define the genetic basis in both murine and bovine hosts behind either susceptibility or relative resistance to infection (termed ‘trypanotolerance’) (3,4). However, an increasing focus of research is how the genetic background of the parasite can also have a significant influence upon the outcome of infection. This is obviously not a new topic for study, as it has long been recognized that there are virulent and less-virulent strains of parasite. Indeed, the human-infective subspecies of *Trypanosoma brucei*, *Trypanosoma brucei gambiense* and *Trypanosoma brucei rhodesiense* are classically described as causing very different diseases, with *T. b. gambiense* causing chronic infections and *T. b. rhodesiense* resulting in acute and severe disease (5). However, although trypanosome strain variation has long been acknowledged as an influence upon disease progress, relatively little attention has been focused upon the genes or gene products in trypanosomes that are responsible for driving disease in the host towards severity or otherwise. It seems clear that to produce a holistic model of the host–parasite interactions that determine infection and disease dynamics, the evident ability of the parasite to modulate these outcomes must be incorporated (6,7). Recent research has started to shed light on trypanosome virulence as a selective and a selected trait and has begun to identify trypanosome genes and gene products that are virulence factors driving disease outcome. This review will concentrate upon those studies that give particular insight into how different trypanosome strains are able to cause different disease outcomes and will highlight potential virulence factors that have been identified to date. How the host specifically responds to trypanosome infections is well covered elsewhere (7–9), including in this issue (see Magez, Namangala and Bucheton), and this will only be discussed with respect to how parasites may be influencing the host response.

What do we mean by a ‘virulent’ trypanosome? This term has been used to describe several different phenotypes and so it is important to define these. In principle, one can consider three broad descriptors: (1) the level of parasitaemia and the prepatent period, with virulent strains being those that give a high parasitaemia and short prepatent period, (2) transmission efficiency by the vector, with virulent strains being those that are transmitted rapidly and give high levels of vector infection, and (3) the level of pathology in the host species (anaemia, organomegaly, tissue penetration, etc.), with virulent strains inducing more severe disease as measured by these parameters. It is uncertain whether these different expressions of virulence are facets of the same phenotype or are distinct but interrelated phenotypes. Intuitively, the latter seems the most likely with different parasite gene products being responsible. Considering a strain that grows quicker than another, this may indeed contribute to more severe disease, but there is often not an absolute correlation between parasite numbers and severity of pathology (10,11). Growth, particularly in trypanosomes, is also a phenotype that can rapidly alter through both *in vivo* and *in vitro* passage, and this instability of phenotype in laboratory conditions can make it an unreliable measurement in terms of strain-specific virulence (12) (although this does not mean that growth cannot inform *per se* regarding pathogenesis, e.g. by examining the basis of growth rate variants isolated from the field). Because of the flexibility of the growth phenotype in trypanosomes, identifying any genetic basis behind it is likely to prove difficult. An added complication is that parasites continuously passaged in mice, and in all likelihood those that are continuously grown in culture, become ‘monomorphic’ and lose the ability to differentiate in a density-dependent manner to the short stumpy life cycle stage (13,14). In the light of this, it is obvious that these parasites will be ‘virulent’, as their lack of self-limiting differentiation will allow continuous exponential growth, and in the mouse model, inevitably rapid death. Therefore, it is essential to use pleomorphic trypanosomes if a realistic model of the disease process is to be analysed. In any case, the situation is often more complex than a simple growth phenotype (15), and perhaps the more interesting and informative scenario is where one strain causes more severe disease than the other, despite having similar growth rates. In these instances, the phenotype can be measured and the contribution of each parasite strain to host phenotypes can be quantified, as well as providing a comparative approach towards identifying the genetic or proteomic basis for the differences. An additional complication in a number of pathogen researches is the use of model hosts (mice) as opposed to ‘natural’ hosts (e.g. livestock or humans for trypanosomes). However, it is becoming increasingly clear that the mouse provides a crucial experimental tool to dissect the processes that are central to pathogenesis in trypanosomiasis (16,17). The post-genomic era has allowed a move away from the focus on a single gene, towards a more holistic identification of pathways, their connections and the common processes between different host models that may prove crucial to our fuller understanding of how the disease manifests and progresses in trypanosomiasis.

The incorporation of parasite virulence into disease models has proved useful for the understanding of infection dynamics in several other protozoan parasites. In particular, the examination of the relative contribution of parasite virulence towards disease has been examined in *Plasmodium* species (18–20), where the implications of parasite virulence and the selective pressures that result in virulence are starting to be understood and examined at both the individual and population levels (21). Different species of *Leishmania* have tropism for different host tissues that results in very different patterns of pathology, and this has been investigated both at the genomic level (22) and with respect to specific genes that are potentially responsible (23). However, it must be stressed that we do not know if these are strain- or species-specific traits. Perhaps one of the most elegant demonstrations of the use of genomic and post-genomic tools to identify a parasite genetic basis of virulence has been carried out for *Toxoplasma gondii*, where using a forward genetic approach a serine-threonine kinase was identified as a virulence factor (24,25), the role of which has since been confirmed in differential virulence in field isolates (26). The impact of this gene during infections in the natural hosts (i.e. cat and man) is perhaps the final piece of this picture remaining to be analysed. These approaches have until recently not been comprehensively used to analyse trypanosomes, in which the understanding of the contribution of the parasite genetic background to disease remains scanty by comparison.

We are beginning to understand how genetic variation in the trypanosome arises, whether it is by clonal or sexual reproduction, and are able to measure the levels of genetic diversity within and between geographically distinct foci of trypanosome disease. The role of sexual recombination and genetic diversity does vary in terms of species; for example, *T. vivax* seems to multiply clonally and remains relatively similar within a focus, whereas *T. congolense* undergoes frequent sexual recombination and is genetically diverse even within a population from a single endemic focus (27,28). The situation in *T. brucei* is more complex, and while the limited number of studies suggest that field populations in nonhuman hosts (e.g. cattle) undergo frequent mating and are genetically diverse, the human-infective subspecies undergo genetic exchange rarely and are genetically less diverse, at least within geographic foci (29–32). Understanding the dynamics of gene flow within populations is important to further comprehend how traits spread spatially and temporally. The level of genetic variation and differentiation that has been described will undoubtedly contribute to phenotypic variation, but we know relatively little about how genetic variation translates into phenotype differences in *T. brucei*, and it is this link between genotype and phenotype in the parasite that is the focus of this review.

This review will consider three aspects of virulence from the parasite perspective, firstly strain-specific variation in virulence, secondly identified candidate virulence genes and thirdly the importance of virulence in epidemiological and evolutionary contexts.

## Identified strain-specific gene effects in *T. brucei*

### Strain variation in virulence

The analysis of differing disease outcome with respect to trypanosome strain or genotype has been examined historically using basic pathology parameters, e.g. (33,34), which provided useful descriptions of strain-specific differences in pathology. More recent studies have begun to elucidate the trypanosome factors responsible for these different pathologies and have addressed the problem using different approaches. Virulence variants of *T. b. gambiense* (tested in mice) from the same focus of disease have been analysed using proteomics to identify components in the trypanosome secretome, with the rationale that secreted trypanosome proteins may be responsible for the key host–parasite interactions influencing disease outcome (11). This study identified both proteins that were differentially expressed between the strains, as well as proteins that were expressed only in one strain or the other. The analysis of the host response suggested that it was macrophage activation that was key to the virulence status of the parasite. The causative proteins in this instance, and whether it is differential or absolute expression of one or several factors, remain to be elucidated however.

More detailed insight into a specific host–parasite interaction has been gained using an experimental model of the blood–brain barrier, where it has been demonstrated that different strains of trypanosome are able to cross the endothelial barrier with different efficiencies; IL1852 (a *T. b. rhodesiense* isolate) is able to cross with much greater efficiency than TREU927 (*T. b. brucei*) (35). The basis of this has been demonstrated to be at least in part because of a secreted cathepsin L cysteine protease, brucipain (36), and is because of alteration of intracellular calcium concentrations in the endothelial cells that is mediated by brucipain (37), perhaps via protease-activated receptors on the host cell (38). Although this has been suggested to provide the human-infective trypanosomes in particular with a mechanism for brain tissue tropism, only one strain of human-infective parasite has thus far been used. Despite this, the study provides clear evidence for a virulence factor. The basis of this differential virulence was correlated with differential activity of brucipain in the secretome, with the *T. b. rhodesiense* lysate having 10-fold greater protease activity than that of *T. b. brucei* (37). The basis behind this differential activity is an important issue to address: is the protease itself more active (is this because of a strain-specific polymorphism, for example) or does the *T. b. rhodesiense* strain secrete more of the protease – as a multicopy gene do gene copy numbers vary between strains – or is it variation at the individual gene expression level? There is some evidence for the latter scenario, as when expression of brucipain was reduced using RNA interference, the ability of *T. b. brucei* to cross an *in vitro* endothelial layer was inhibited by 50% (36). Given the potential importance of this virulence factor, these questions are a significant area of interest for understanding strain-specific virulence. Although previous attempts to use the *T. congolense* orthologue, congopain, as an antidisease vaccine candidate in cattle met with a degree of success (39), *T. congolense* is largely an intravascular parasite, whereas *T. brucei* is extensively extravascular, and therefore it could be postulated that virulence factors such as brucipain in *T. brucei* will consequently have more disseminated and tissue-specific effects (as an obvious example, involvement of the brain and sleeping sickness).

### Forward genetics approach to trypanosome virulence variation

In our laboratory, we have been using a forward genetic approach to identify regions of the *Trypanosoma brucei* genome that contain genes that are responsible for differential pathogenesis. This provides an unbiased approach to identify the causative gene(s), because no *a priori* hypothesis of putative responsible factors needs to be invoked. Two strains of *T. brucei* form the basis of these studies, TREU927 (the genome reference strain) and STIB247. These strains differ inherently in the pathology that they induce during infections in mice (15). It should be emphasized that the differences are not because of the parasites simply having different growth rates, and the strains used in our study were matched for passage numbers and crucially were both pleomorphic parasites that have maintained the key characteristics of ‘wild-type’ trypanosomes (differentiation to the short stumpy life cycle stage and tsetse transmissibility). Inbred (BALB/c) mice infected with either STIB247 or TREU927 displayed significantly different pathology, with TREU927 infections resulting in more severe anaemia, reduced reticulocytosis, a less severe degree of splenomegaly, and increased IFNγ and IL-10, but reduced IL-12, levels compared to STIB247 infections (when measured up to day 12 post-infection). Gene expression analysis of host cells in the spleen at day 10 post-infection was undertaken (15), and pathway analysis revealed that the main processes that were differentially regulated between the infections were Liver X receptor activation, IL-10 signalling and alternative macrophage activation. It should be noted that pathways linked to the acquired immune response were significantly upregulated in both infections, but to the same degree, and it was therefore considered unlikely that they were responsible for the differential pathogenesis. These data combine to suggest that the innate immune response is being differentially activated by the parasite strains and therefore that there must be a significant genetic basis in the parasite for this phenomenon.

These two strains had previously been used to generate the first genetic map of *T. brucei* (40). The availability of this and accompanying resources allowed the use of a classical forward genetic approach to analyse the inheritance of the pathogenesis phenotypes in infections with the F1 progeny clones that had been generated and incorporated in the genetic map (41,42). Infections were undertaken in BALB/c mice with the progeny clones and the phenotypes measured as in Morrison *et al.* (2010). Quantitative trait loci (QTL) analysis was undertaken to identify regions of the genome that contained genes responsible for the measured traits (43). A QTL on *T. brucei* chromosome 3 (LOD score = 7·2), which contributed approximately 65% of the variance observed in both splenomegaly and hepatomegaly, was the major finding of the study. The QTL encompasses 383 genes, and so further work is required to identify the gene(s) responsible within the QTL interval. This can be approached from several angles: firstly, fine mapping to reduce the interval boundaries by using additional informative markers [microsatellites and single nucleotide polymorphisms (SNPs)] and through the isolation of further progeny clones with informative crossovers; secondly, a more defined phenotype will provide more information – i.e. the measurements used (spleen and liver weight) can undoubtedly be influenced by multiple mechanisms and pathways, but by using, for example the expression levels of key genes in the implicated pathways (15), this may give much cleaner segregation patterns and significant linkage to a smaller region of the chromosome; and thirdly by identifying candidate genes [for example, by SNP analysis – the gene must be heterozygous in TREU927 to be informative in this cross (15)]. In addition, a second, less significant, locus on chromosome 2 contributed to splenomegaly, hepatomegaly and reticulocytosis. The importance of these findings is that this provides an avenue to identify genes in the trypanosome genome that are responsible for influencing the host immune response and determining the severity of infection, and this work is ongoing. These findings have parallels with field data, where genotypic differences in Ugandan *T. b. rhodesiense* isolates correlated with differing severity of disease in sleeping sickness patients (44,45) – although this conclusion does require further confirmation, because of the inherent difficulty of eliminating host variation as a confounding factor.

## Candidate virulence factors

It seems clear from studies into how the host responds to trypanosome infection that several facets of the innate immune response are crucial in determining disease outcome. In particular, the polarization of the immune response, whether it is IFNγ or IL-10 dominated (46,47), or whether alternative or classical macrophages are predominant (48,49), together with the role of regulatory T cells (50–52), seem to be crucial in shaping the severity and duration of the pathology observed. Within this, a broad conclusion can be drawn from studies using hosts of differing inherent susceptibility or tolerance to trypanosomes (10,53,54), as well as our study involving different parasite strains (15), that it is the relative timing of these processes that ultimately determine whether the pathology will be severe or not. Therefore, any parasite gene or gene product that influences the direction in which the activation of the immune response progresses is a candidate virulence factor, and particularly if the expression or activity of the candidate gene varies between strains. As these processes largely involve effector molecules, and tend to be downstream of regulatory processes, it seems reasonable to assume that it will be parasite-based differences in activation at the recognition or regulatory stage, for example pattern recognition receptors such as Toll-like receptors, that initiate and are responsible for the direction of polarization of downstream effector arms of the response. As yet, the parasite products that directly affect the features of the host immune response have not been characterized for any strain-specific ability to cause different disease outcomes, but several have been implicated as virulence factors *per se* (55) at the single-strain level. For example, in addition to the cysteine peptidase described earlier, other peptidases have been identified that also retain activity in the serum of the infected host, and which therefore may be able to effect host processes directly (56,57). Perhaps the best characterized immunostimulatory parasite product is the variant surface glycoprotein (VSG), which is the mainstay of the trypanosome's remarkable system of antigenic variation (58–60). Aside from their well-characterized role as changing cloaks that disguise the trypanosome from the host acquired immune response, the glycosylphosphatidylinositol anchors that tether the VSG to the parasite surface membrane have been shown to be a key factor in immune response activation; this is particularly the case with soluble VSG, which is actively cleaved by parasite phospholipase-C and released into the extracellular medium. Both the glycosylinositolphosphate (on the shed VSG) and dymyristoylglycerol (membrane bound) moieties have been implicated in shaping the immune response to trypanosomes (61–63). How the VSG and associated moieties could result in differential disease outcome based on strain-specific differences is unclear, but it could be that different strains exhibit structural differences, including the possibility of variable polysaccharide side chains, or different rates of VSG shedding, which may lead to variable activation of host receptors. Intriguingly, a trypanosome protein that directly modulates the host's immune response, termed the trypanosome-suppressive immunomodulating factor (TSIF) was recently described (64). The protein was suggested to be a surface-bound membrane protein, inferred by the fact that antibody raised against recombinant TSIF-stained fixed cells, but not live cells, and this was confirmed using immunoprecipitation from lsyates of parasites that were surface-labelled with I^125^. Recombinant TSIF induced TNFα and NO production from macrophages *in vitro* and reduced type 2 immune response-mediated pathology *in vivo*. This suggests that TSIF activates TNFα-producing classically (M1) activated macrophages. Although this seems apparently paradoxical at first glance, as type 1 immune responses and IFNγ production are correlated with control of parasite numbers (46), the authors suggested that the activity of TSIF is consistent with it playing a role in the immunosuppression that is characteristic of trypanosome infections (65,66). An intriguing prospect would be to analyse TSIF from different strains of trypanosome and characterize any differential effect.

Although we are increasing our understanding of the host immunological forces that respond to trypanosome infection and shape the disease process, it is clear that in this context the contribution of the trypanosome is less well understood. The examples described here demonstrate that candidates have been identified for parasite products that influence the host response, but most of these have not been examined with respect to the range of disease patterns caused by different strains of the parasite. Additionally, commonalities from several studies, for example the activation status of macrophages as being a crucial indicator of the virulence of a trypanosome strain (11,15,64), suggest that in addition to providing insight into how the parasite causes disease, parasite virulence variation could well prove to be an invaluable tool for dissecting the host response to trypanosome infection.

## Importance and epidemiological implications

A crucial perspective when considering virulence is why do parasites evolve to become virulent or less virulent – what are the selective pressures that drive parasites to become more or less pathogenic? One obvious variable that has been studied is the influence of different host genetic backgrounds, as has been suggested as a key factor for natural infections with *T. congolense* (67). This has been proposed as a reason why virulent parasite strains of *T. congolense* emerge in regions where different host species predominate in Zambia – relatively susceptible cattle populations and relatively resistant wild animals presenting different selective pressures on parasite virulence (67). It seems clear that there will be a two-way relationship in this context, with virulence of the parasite and tolerance of the host imposing selection, the selective pressure being dependent upon the status of each participant. An area that has been extensively studied in *Plasmodium* is the relationship between parasite virulence and transmission to the vector, and the evolutionary trade-offs between harm to the host and overall transmission fitness (19). Put simply, the ultimate objective of a mammalian infection from the trypanosome perspective has to be successful transmission onwards to the tsetse vector. Recent work has postulated, based on data obtained from studying the protozoan parasite *Ophyrocystis elektroscirrha* of the monarch butterfly, that the ‘optimum’ virulence state of a parasite in terms of balancing harm to the host with successful transmission is intermediate virulence (68). However, this is again looking at one side of a coin – if different selective pressures from the host are considered, then the optimum virulence of the parasite for eventual transmission becomes relative to the susceptibility/resistance of the host. This was confirmed in a follow-up study, where the virulence status rankings for *O. elektroscirrha* genotypes differed between host genotypes, and the authors concluded that it is crucial to consider genotype–genotype interactions when examining the evolution of virulence in a larger context (69). Therefore, it becomes apparent that the pathogenic status of a parasite becomes intrinsically linked to the selective pressures that are imposed by the genetics of the other integral components of the holistic life cycle. With vector-borne parasites, this includes the often forgotten potential selective effects within the vector, as has been proposed in *Theileria* and analysed in *Plasmodium*, where genetic bottlenecks in the arthropod vector have been suggested to play a role in the pathological outcome in the mammal (70,71). Similar selective bottlenecks have recently been demonstrated to occur in the tsetse vector with *T. brucei* (72). An additional level of complexity that needs to be considered is the influence of mixed infections, whether with more than one species of trypanosome or other parasite (73) or from co-infection with different strains of the same parasite (74,75) – both of these scenarios undoubtedly occur widely in the field. Thus, to understand the evolution of pathogenesis/virulence in trypanosomes, one must do so in an integrated fashion, incorporating genetic and phenotypic variation in the trypanosome, the mammalian host and the tsetse vector, otherwise any findings will only have relevance for the particular genotype–genotype interaction being considered. These aspects of the relationship between host, vector and parasite have been relatively understudied in trypanosomes, but the availability of defined trypanosome strains, in addition to the well-characterized mouse strains, means that the tools are now available to address these questions.

## Conclusions

How the parasite itself shapes the course of disease is a relatively neglected but important area of research with respect to trypanosomes. Identifying how virulent strains cause clinical signs will increase our understanding of how disease progresses, addressing a fundamental question of trypanosome biology, and provide the potential to develop antidisease strategies. The post-genomic tools now available provide an incredible opportunity to begin to understand these processes. The field is moving on from single-gene studies towards focussing on identifying regulatory genes and processes, by taking advantage of technological advances such as RNAseq, the ability to visualize *in vivo* and live cell–cell interactions (76), multiple pathogen and host genome sequences, the impending genome sequence of the tsetse vector, *in silico* modelling, and the increasing number of gene knockout mice available. Therefore, the opportunity now exists to incorporate these approaches in a more systems-based manner, enabling us to understand more completely the bigger picture and the unravelling of how the key host–parasite interactions drive disease. Crucially, the introduction of parasite genetic and phenotypic variation into existing disease models of trypanosomiasis will provide experimental resources to allow a deeper understanding of both host and parasite determination of disease outcome and the addressing of larger evolutionary questions.
